# Highlights from the 1st ISCB Latin American Student Council Symposium 2014

**DOI:** 10.1186/1471-2105-16-S8-A1

**Published:** 2015-04-30

**Authors:** R Gonzalo Parra, Franco L Simonetti, Marcia A Hasenahuer, Gabriel J Olguin-Orellana, Avinash K Shanmugam

**Affiliations:** 1 Protein Physiology Lab, Departamento de Química Biológica IQUIBICEN-CONICET, Facultad de Ciencias Exactas y Naturales, Universidad de Buenos Aires, Argentina; 2 Fundacion Instituto Leloir, Universidad de Buenos Aires, Argentina; 3 Structural Bioinformatics Group, Universidad Nacional de Quilmes, Argentina; 4 Laboratory of Structural Biology and Nanophysiology of Pontificia Universidad Católica de Chile, Chile; 5 Department of Computational Medicine and Bioinformatics, University of Michigan, Ann Arbor, USA

## Abstract

This report summarizes the scientific content and activities of the first edition of the Latin American Symposium organized by the Student Council of the International Society for Computational Biology (ISCB), held in conjunction with the Third Latin American conference from the International Society for Computational Biology (ISCB-LA 2014) in Belo Horizonte, Brazil, on October 27, 2014.

## About the Student Council and the symposium

The Student Council (SC), part of the International Society for Computational Biology (ISCB), aims at nurturing and assisting the next generation of computational biologists. Our membership and leadership are composed of volunteer students, post-docs and young professionals in bioinformatics, computational biology and related fields. The main goal of our organization is to offer networking and soft skill development opportunities to our members.

The Latin American Student Council Symposium (LA-SCS) 2014 represents the first edition of this symposium. Organization of this event drew up on experiences of the Student Council in successfully organizing the Student Council Symposium (a satellite symposium to the ISMB conference) every year for the past ten years [1-8] and three editions of the European Student Council Symposium [9] (a satellite symposium to the ECCB conference). The LA-SCS is planned to be organized every other year, directly preceding the ISCB-Latin America conference, with the next one to be organized at Buenos Aires, Argentina in 2016.

## Scope and format of the meeting

The Latin American Student Council Symposium is a day-long meeting held in conjunction with the Latin American conference from the International Society for Computational Biology (ISCB-LA) that is organized bi-annually. The main goal of this symposium is to gather together students in the field. The Bioinformatics and Computational Biology fields have been growing steadily in the last years and the Student Council wants to accompany this process by nurturing the next generation of bioinformaticians and computational biologists. The Student Council achieves this by creating opportunities to meet peers from all over the world, promote the exchange of ideas, provide networking opportunities and giving travel fellowships to its members in order to attend the different events that are organized.

The traditional scientific component of the LA-SCS meeting consisted of three sessions, each with a keynote talk and several student presentations. During the lunch and in the evening, everybody got the opportunity to present their work during the poster sessions.

On this occasion, we were honored with excellent keynote lectures that were kindly delivered by Prof. Vitor Leite (Universidade Estadual Paulista, Brazil), Prof. Francisco Melo (Pontificia Universidad Católica de Chile), and Prof. Peter F. Stadler (University of Leipzig, Germany).

The symposium received 103 submissions from students. These submissions were peer-reviewed by 16 independent reviewers. 7 abstracts were selected for oral presentation and 34 additional abstracts were accepted for poster presentations. Abstracts from the oral presentations and the abstract awarded best poster are included in this meeting report. Abstracts of the other poster presentations are available online in the symposium booklet (http://lascs2014.iscbsc.org/lascs2014-booklet).

## Keynotes

Keynote presentations form a fundamental part of these meetings, giving students the opportunity to interact with and learn from top researchers in the field.

Our first keynote speaker, Prof. Leite, delivered a lecture entitled “Protein Folding Funnels and Applications to Bioethanol Production” in which he explained how theoretical approaches to protein folding (Energy Landscapes Theory and the Minimal Frustration Principle), were useful to modify biomolecules and improve industrial processes with high economic impact, such as bioethanol production. Prof. Leite explained in detail the theoretical framework of this work as well as the many tools developed at his group in order to deal with and analyse the structural data from proteins.

Prof. Melo presented the second keynote lecture, entitled “Development of new bioinformatics tools to study the key molecular determinants that mediate protein-DNA recognition”, in which he described several bioinformatics tools that were developed at his group that are useful for the study of protein-DNA interactions. Structural databases, knowledge-based potentials, Metropolis-Montecarlo simulations and Pymol plugins were some of the tools developed by Prof. Melo and presented at this talk.

For the last keynote Prof. Peter F. Stadler presented to us a lecture on “Phylogenetics from Paralogs”. Sequence-based phylogenetic approaches rely on initial data sets to be composed of orthologous sequences only. Based on mathematical phylogenetics, Prof. Stadler’s group demonstrated that plausible phylogenetic trees can be inferred from paralogy information too. While the resolution is very poor for individual gene families, genome-wide datasets are sufficient to generate fully resolved phylogenetic trees of several groups of eubacteria.

## Student presentations

Delivering the first student presentation, Guimarães et al. [10] presented Mendel,MD an efficient, secure and reliable software to explore variants from exome data of patients with Mendelian disorders. While the software is efficient and sophisticated from the computational point-of-view at the same time it is simple and user-friendly for clinicians to quickly analyse genomic data.

Biomining, by using acidophilic and chemolithotrophic microbes to recover metals of interest from complex minerals, generates acid mine drainage (AMD) that pollutes water and sediments with acids and metals. Medeiros et al. [11] assessed the taxonomic and functional microbial diversity in a mining area in the Brazilian Amazon in order to find alternative microbes to bioremediate AMD contamination.

Sifter v2.0 is considered as one of the best tools for protein functional annotation with an approach that combines phylogenomics and bayesian graphical models. However due to usability and suitability issues to perform high-throughput analysis, the software is not widely used yet. Almeida-E-Silva et. al. [12] presented Sifter-T a new implementation that aims to maintain the strengths while tackling the weaknesses of this software.

Gap Junction Channels (GJCs) connect the cytoplasm of adjacent cells, providing a hydrophilic path between cells that allow the movement of different small molecules through passive diffusion. Escalona et al. [13] built several models and performed molecular dynamics simulations applying uniform external electric fields in order to understand the voltage effects and ion transport mechanisms at GJCs.

Protein subfamily classification commonly relies on grouping proteins according to their sequence similarities. However, there is no single sequence similarity threshold that serves to accurately group proteins into isofunctional groups. Simonetti et al. [14] showed us that the use of coevolution between aminoacids at sequence alignments may prove a useful tool to aid in the problem of protein subfamily classification.

It has been accepted that natural foldable proteins are selected through natural selection, satisfying the minimum frustration criterion (minimizing their internal energetic conflicts). However, remaining frustration, i.e energetic conflicts, can have functional consequences. Contesotto et al. [15] analysed the effect of topological and energetic frustration on protein folding speed by applying structure-based-models simulations.

Finally, Unspliced EST data have received little attention as a source of information on non-coding RNAs. A subclass of the unspliced EST cluster consists of so called 3'UTR-derived RNAs (uaRNAs). Engelhardt et al. [16] talked about the evolution of this type of RNAs that can have functions both in cis and trans, independent from the harboring gene, and presented candidates for further experimental verification.

## Award winners

Based on the votes of the LA-SCS organizing committee, winners for the two best oral presentation awards and a best poster award were chosen. Among oral presentations the first place went to Raony Guimarães, for his work “Mendel,MD: a user-friendly online program for clinical exome analysis” [10]. The second place oral talk was by Danilo Almeida-E-Silva, for his work “Sifter-T: A scalable framework for phylogenomic probabilistic protein domain functional annotation” [12]. Javier Caceres’ work on “Rational discovery of new capsaicin analogues as TRPV1 activators” [17] was adjudged the best poster.

## Workshop

Apart from the main event we also implemented at LA-SCS a new idea, to offer introductory bioinformatics workshops designed and taught by Student Council members. Given the natural heterogeneity of undergraduate backgrounds in our students we think it important to take advantage of the complementarity among their different profiles and backgrounds. Thus PhD/post-doc students from the computer science backgrounds can offer basic one-day courses to those students coming from the biological careers and vice-versa. These courses are planned to be held as satellite events before or after the symposium day. R. Gonzalo Parra and Franco L. Simonetti, two PhD students from the Regional Student Group Argentina (RSG-Argentina) organized and presented a workshop entitled “Intro to BioPerl for Pipeline building in Bioinformatics” that was held on October 31^st^. The eight hour workshop included a general introduction of the Perl language, presented along with examples and documentation for the students, and a second half introducing the BioPerl project and other tools such as CPAN modules, GnuPlot and interconnecting these tools in order to build quick and useful pipelines.

## Conclusions

The organization of a first time symposium in a region faces several difficulties: lack of an integrated network of long term student council members to constitute the committees and difficulties to attract sponsors just to name two. However, after several calls through social media and universities mailing lists, we were able to create a group of enthusiastic local volunteers that little by little were feeling as important parts of an exciting project. After months of planning we were able to recruit high quality keynote speakers, a good number of abstract submissions and even some money from sponsors! Finally, the LA-SCS, in its first year, had a comparable number of attendees to the more established European and American editions of the Student Council Symposium 2014.

The next edition of this meeting will be held in Buenos Aires, Argentina. We still have a long road ahead us, many things to improve and many failures to face but we are confident that we have created a good foundation that will allow the next group of students from the region leading LA-SCS 2016 to take it to greater heights. For further information regarding the Student Council, its events, internships and community, please visit http://www.iscbsc.org
